# Non‐invasive ventilation treatment for patients with chronic obstructive pulmonary disease

**DOI:** 10.1049/htl2.12048

**Published:** 2023-06-08

**Authors:** Fleur T. Tehrani, James H. Roum

**Affiliations:** ^1^ Department of Electrical and Computer Engineering California State University Fullerton California USA; ^2^ School of Medicine University of California Irvine California USA

**Keywords:** decision support systems, lung, medical expert systems, oxygen, patient treatment, physiological models, ventilation

## Abstract

Chronic obstructive pulmonary disease (COPD) affects the lives of millions of patients worldwide. Patients with advanced COPD may require non‐invasive ventilation (NIV) to support the resultant deficiencies of the respiratory system. The purpose of this study was to evaluate the effects of varying the continuous positive airway pressure (CPAP) and oxygen supplementation components of NIV on simulated COPD patients by using an established and detailed model of the human respiratory system. The model used in the study simulates features of advanced COPD including the effects on the changes in ventilation control, increases in respiratory dead space and airway resistance, and the acid–base shifts in the blood seen in these patients over time. The results of the study have been compared with and found to be in general agreement with available clinical data. Our results demonstrate that under non‐emergency conditions, low levels of oxygen supplementation combined with low levels of CPAP therapy seem to improve hypoxemia and hypercapnia in the model, whereas prolonged high‐level CPAP and moderate‐to‐high levels of oxygen supplementation do not. The authors conclude that such modelling may be useful to help guide beneficial interventions for COPD patients using NIV.

## Graphical abstract

• Optimal application of Non‐Invasive Ventilation (NIV) can help mitigate the negative impacts of Chronic Obstructive Pulmonary Disease (COPD).

• Optimal applications of continuous positive airway pressure (CPAP) component of NIV can improve oxygenation and reduce carbon dioxide retention for COPD patients.

• Optimal applications of supplemental oxygen treatment component of NIV can result in normoxia and reduced hypercapnia in COPD patients.

• Mathematical modelling and computerized decision support systems can be used to help guide beneficial interventions for COPD patients using NIV.

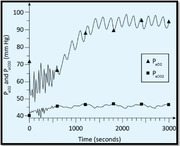

## 1 INTRODUCTION

Chronic obstructive pulmonary disease (COPD) is a respiratory disorder exhibiting features of emphysema and chronic bronchitis which causes respiratory system disability and millions of deaths every year worldwide and can lead to mechanical ventilation dependence in advanced cases [1, 2, 3, 4]. While the precise etiology of the disease is not yet clearly understood, some factors like smoking, repeated respiratory infections, childhood pulmonary diseases like asthma, and genetic factors have been identified as predisposing contributing factors [3, 5–6]. In COPD, alveoli become damaged over time, affecting gas exchange in the lungs and altering the ventilation/perfusion ratio [7, 8, 9]. In addition, the bronchi become inflamed and disfigured, leading to increases in respiratory airway resistance and work of breathing. These conditions lead to chronic hypoxemia and hypercapnia that in turn cause acid–base shift in the blood and the cerebrospinal fluid (CSF) [2, 4], alter the response of the respiratory control system to various stimuli, and affect the functions of all the body organs. Among different treatment options, long‐term use of non‐invasive ventilation (NIV), which can provide continuous positive airway pressure (CPAP) and supplemental oxygen treatment, has been proposed in various studies to mitigate the negative impacts of the disease and improve survival among COPD patients [10, 11, 12, 13, 14, 15, 16].

In a review article on the use of NIV for COPD patients [17], it was concluded that using home NIV treatment may reduce the mortality rate of patients afflicted by COPD. In another study, the long‐term effects of the use of NIV for COPD patients were investigated [18]. It was concluded that long‐term nocturnal NIV can be beneficial for COPD patients. However, more studies on optimal management of NIV in COPD were considered as necessary.

Nowadays, mathematical models are used increasingly and extensively to study the functions of different body organs and to develop therapeutic devices and medications to treat various diseases. The purpose of this study was to use a detailed mathematical model of the human respiratory system in COPD [19] to help find an optimal approach for the management of NIV treatment for COPD patients under non‐emergency conditions. This study is used to anticipate the effects of CPAP therapy and oxygen supplementation components of NIV on COPD patients. The simulation results have been compared with available clinical observations in the literature. A brief overview of the model used in the study is provided in the following section.

## 2 METHODS

**FIGURE 1:**
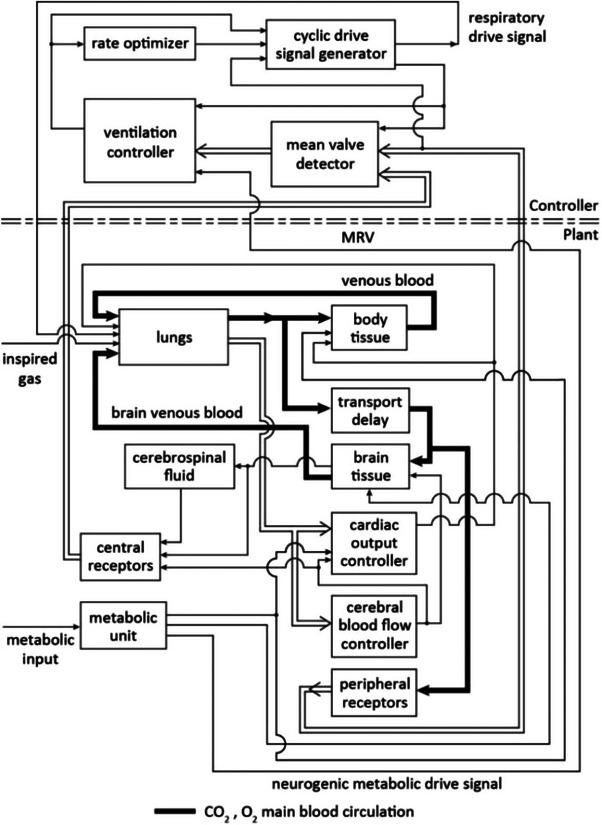
A block diagram of the model used in the study [19].

The mathematical model used in this study [19] is a modified version of an earlier detailed model of the human respiratory system [20, 21]. The modifications are particularly tailored to better simulate COPD patients. Figure 1 shows a block diagram of this model.

As shown in Figure 1, the model consists of a continuous plant and a discrete controller. The controller updates and generates a respiratory drive signal for every breath. A ventilation controller determines the required ventilation based on the chemical composition of arterial blood and the blood at the site of the central respiratory receptors for every breath. A ‘rate optimizer’ receives the required ventilation data and data on the mechanical properties of the respiratory system and updates the optimal respiration rate for a next breath to minimize the respiratory work rate. A drive signal is generated by the respiratory controller and sent to the lungs that is updated for every breath.

The plant in Figure 1 includes ‘lungs’, ‘body tissue’, ‘brain tissue’, a ‘CSF’ compartment, a ‘transport delay’, that simulates the delay in arterial transport from the heart to the brain, ‘peripheral receptors’, ‘central receptors’, and a ‘metabolic unit’, that produces a metabolic drive signal to the respiratory control system. The plant also includes a ‘cardiac output controller’, and a ‘cerebral blood flow controller’, that determine the cardiac output and the cerebral blood flow as dynamic functions of the patient's arterial blood gases and metabolic rate continuously. The model includes the effects of reduction in the ventilation/perfusion ratio in COPD, increase in the respiratory dead space under the disease conditions, and the increase of the work of breathing and the airway resistance. In COPD, the bicarbonate levels of the arterial blood and the CSF are elevated to compensate for chronic hypercapnia [2]. The following equations are used in the model [19] to find the hydrogen ion concentration of the arterial blood (H’_a_^+^), the hydrogen ion concentration of the CSF (H_CSF_^+^), and the hydrogen ion concentration of the blood at the site of the central receptors (H_C_^+^):

(1) $$H_{a}^{\prime }=\frac{\left[0.65.{P^{\prime }}_{\mathrm{aCO}2\left(\mathrm{mean}\right)}+13.5\right]}{\gamma }$$

(2) $$H_{\mathrm{CSF}}^{+}=\left[\frac{\alpha \cdot \beta }{{\left(\mathrm{BHCO}3\right)}_{\mathrm{CSF}}}\right]\cdot \frac{P_{\mathrm{CSFCO}2}}{\gamma }$$

(3) $$H_{C}^{+}=\left[\frac{\alpha \cdot \beta }{{\left(\mathrm{BHCO}3\right)}_{\mathrm{CSF}}}\right]\cdot \frac{P_{\mathrm{CCO}2}}{\gamma }$$

where *P*’_aCO2(mean)_ is the mean arterial partial pressure of carbon dioxide, (BHCO_3_)_CSF_ is the bicarbonate concentration of the CSF, and *P*_CSFCO2_ and *P*_CCO2_ are respectively the partial pressures of carbon dioxide in the CSF and at the neighbourhood of central receptors. In these equations, α is the solubility factor for carbon dioxide in CSF (with a typical value of 6.783 × 10^−4^ lit(STPD)/lit/mm Hg), β is the carbonic acid dissociation constant in CSF (with a typical value of 795 nmoles/L), and γ is an illness severity factor greater than one (eg, between 1.2 and 1.7) that indicates the level of rise in the bicarbonate level in the blood in COPD and γ can be used as an indicator of the advancement of the disease over time.

The controller equations of the model are modified to include the effects of a major shift in the acid–base balance that occurs under prolonged disease conditions. The detailed description of this model and the mathematical equations of its plant and the controller can be found elsewhere [19] and, for the sake of brevity, are not given here. However, the ventilation controller equation that includes the COPD effects is the following:

(4) $$\begin{array}{ccc} \mathrm{AVR} & = & \left[0.131+\frac{0.0715}{\gamma }\right]\cdot P{{}^{\prime }}_{\mathrm{aCO}2\left(\mathrm{mean}\right)}+\left[0.131+\frac{0.10227}{\gamma }\right]\\ & & \cdot P_{\mathrm{CCO}2}+\mathrm{FACTV}+\left[-K+\frac{1.485}{\gamma }\right]+\mathrm{MRV} \end{array}$$

In this equation, AVR is the ratio of alveolar ventilation to the normal resting alveolar ventilation, MRV is a neurogenic stimulus drive signal to ventilation which is produced by a metabolic unit in exercise in the model, *K* is a controller constant (with a typical value of 18.885), and FACTV is given by the following equations:

$$\begin{array}{ccc} \mathrm{FACTV} & = & 4.72\times 10^{-9}{\left(104-{P^{\prime }}_{\mathrm{aO}2(\mathrm{mean})}\right)}^{4.9}\mathrm{for}P_{\mathrm{aO}2(\mathrm{mean})}^{\prime }\\ & & <104\;\mathrm{mm}\;\mathrm{Hg} \end{array}$$

and

(5) $$\mathrm{FACTV}=0\;\mathrm{for}\;P_{{}_{\mathrm{aO}2(\mathrm{mean})}}^{\prime }\geq 104\;\mathrm{mm}\;\mathrm{Hg}$$

where *P*’_aO2(mean)_ is the mean arterial partial pressure of oxygen.

In using Equation (4), if AVR becomes negative or if *P*’_aCO2(mean)_ < 33 mm Hg, AVR is set to zero.

The model of Figure 1 was used to simulate the effects of CPAP therapy and various levels of oxygen supplementation in moderate and more advanced levels of COPD. Two stages of the disease were simulated in many experiments and the results were compared with the clinical observations reported in the literature.

## 3 RESULTS

The mathematical model was tested at two different stages of COPD. Table 1 shows the steady‐state values of several selected variables of the model under different test conditions.

**TABLE 1:** The steady‐state results of the model.

	Settings		Results						
COPD stage	CPAP cmH_2_O	*F*_IO2_	*P*_aCO2_ mm Hg	*P*_aO2_ mm Hg	*S*_aO2_	Q L/min	*Q*_B_ L/min	*V*_E_ L/min	f breaths/min
Moderate COPD γ = 1.35	0	0.21	46.0	65.8	0.905	6.7	0.96	5.9	11.0
	0	0.25	46.6	90.0	0.97	6.13	0.94	6.26	11.5
	0	0.3	46.7	127.0	0.994	6.0	0.94	5.8	10.7
	5	0.21	45.8	66.3	0.908	6.5	0.95	5.1	9.8
	5	0.25	46.4	91.0	0.97	6.06	0.92	6.0	11.5
	5	0.3	46.5	131.0	0.995	5.97	0.95	6.04	11.2
	8	0.21	45.8	67.4	0.92	6.5	0.95	5.67	10.7
	8	0.25	46.9	92.17	0.97	6.0	0.95	5.98	11.12
	8	0.3	46.8	126.2	0.994	6.0	0.92	6.3	11.66
Advanced COPD γ = 1.6	0	0.21	48.7	59.2	0.85	7.5	1.32	5.54	10.5
	0	0.25	50.3	87.0	0.96	6.9	1.34	5.73	10.8
	0	0.3	50.14	123.1	0.99	6.9	1.33	5.25	10.1
	5	0.21	48.57	62.0	0.87	7.2	1.26	5.22	10.0
	5	0.25	50.16	90.5	0.964	6.9	1.35	5.5	10.5
	5	0.3	50.28	121.8	0.99	6.9	1.33	5.9	11.0
	8	0.21	48.6	60.5	0.86	7.2	1.30	4.9	9.5
	8	0.25	50.2	86.4	0.956	6.9	1.30	5.9	11.0
	8	0.3	50.2	126.2	0.993	6.9	1.30	5.44	10.3

In the simulation results of Table 1, the steady‐state values were obtained after running the model for 50 min of simulated time under the test conditions so the transient dynamic responses would no longer be present. In this table, *F*_IO2_ is the fraction of inspired oxygen, *P*_aO2_ and *P*_aCO2_ are the arterial partial pressures of oxygen and carbon dioxide respectively in mm Hg, *f* is the breathing rate in breaths/min, *V*_E_, is the minute ventilation in litres, *Q*, and *Q*_B_ are the cardiac output and the cerebral blood flow rate respectively in L/min, and *S*_aO2_ is the arterial oxygen saturation. In the simulation experiments, the respiratory elastance was 34 cmH_2_O/lit, the airway resistance was 12 cmH_2_O/lit/s, and the dead space was increased by 20% above normal. Two stages of COPD are considered. Stage 1 represents a moderate level of the disease while stage 2 represents a more advanced level of the illness. The factor γ in the ventilation controller that indicates the extent of acid–base shifting in COPD is 1.35 in moderate COPD, stage 1 and is 1.6 in more advanced COPD, stage 2.

**FIGURE 2:**
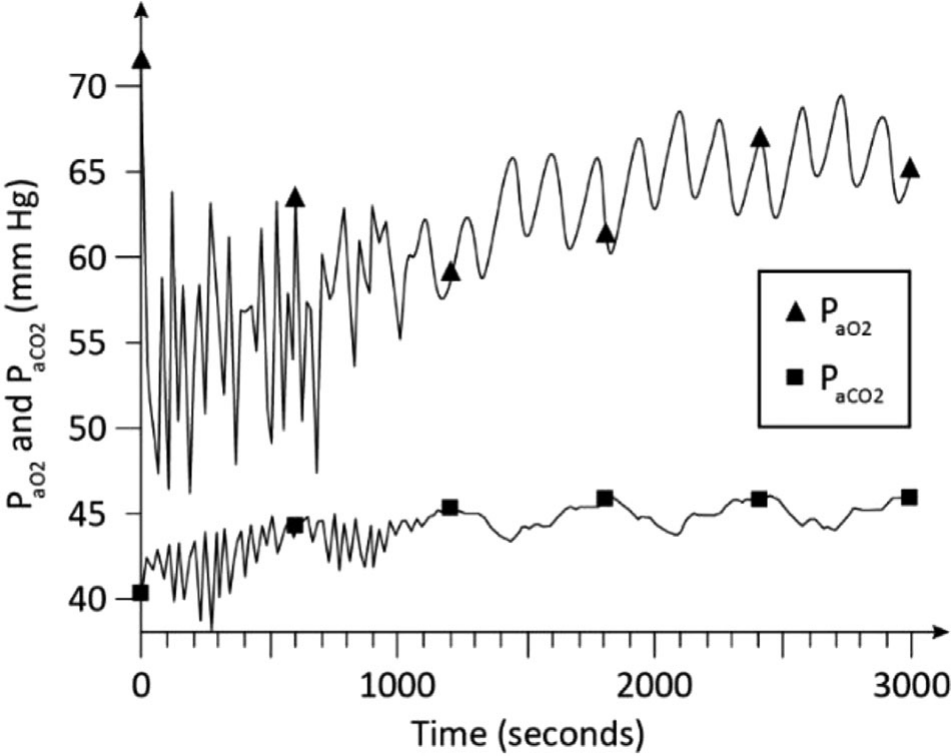
Simulation results in moderate chronic obstructive pulmonary disease (COPD). No continuous positive airway pressure (CPAP) treatment and no oxygen supplementation is used in the experiment. COPD, chronic obstructive pulmonary disease; CPAP, continuous positive airway pressure.

**FIGURE 3:**
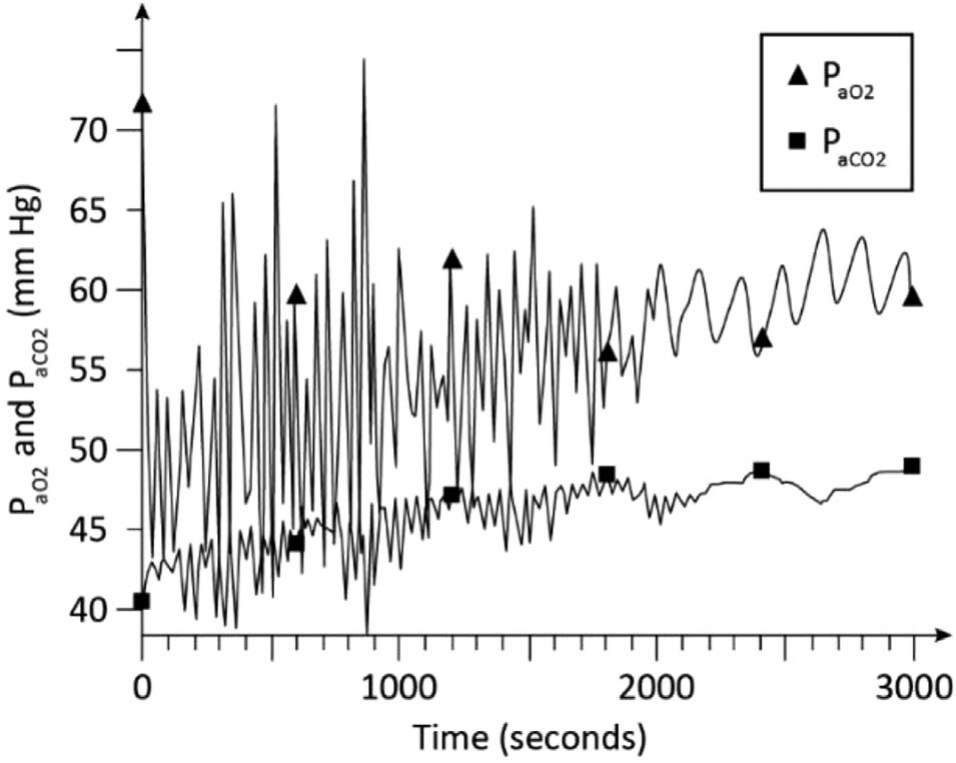
Simulation results in advanced COPD with no CPAP and no oxygen supplementation. COPD, chronic obstructive pulmonary disease; CPAP, continuous positive airway pressure.

**FIGURE 4:**
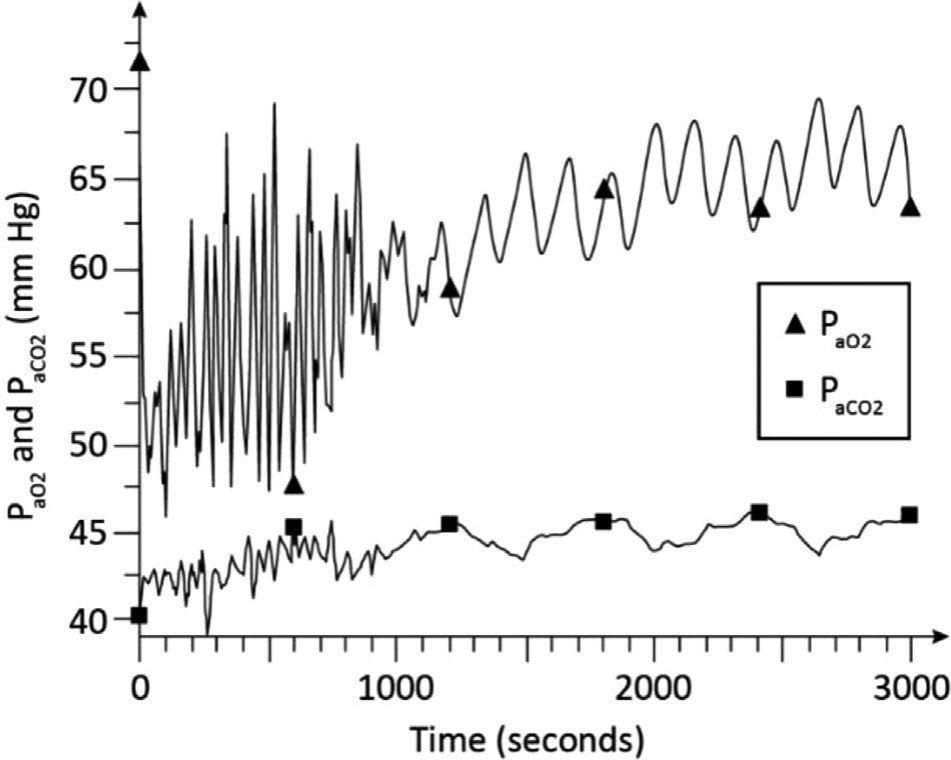
Simulation results in moderate COPD. CPAP = 5 cmH_2_O with no oxygen supplementation (*F*_IO2_ = 0.21). COPD, chronic obstructive pulmonary disease; CPAP, continuous positive airway pressure.

**FIGURE 5:**
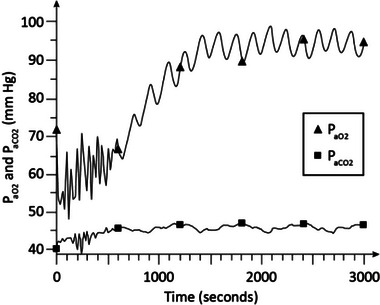
Simulation results in moderate COPD. CPAP = 5 cmH_2_O and *F*_IO2_ = 0.25.

**FIGURE 6:**
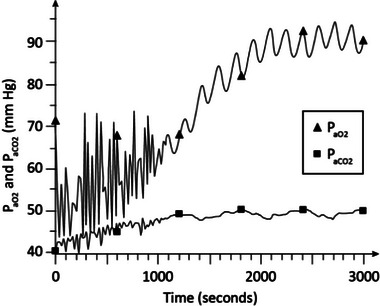
Simulation results in advanced COPD. CPAP = 5 cmH_2_O and *F*_IO2_ = 0.25. COPD, chronic obstructive pulmonary disease; CPAP, continuous positive airway pressure.

**FIGURE 7:**
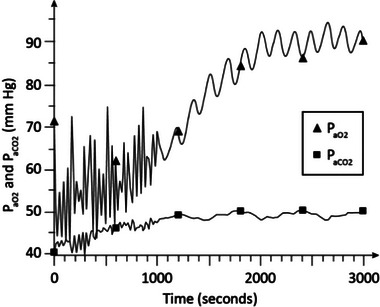
Simulation results in advanced COPD. CPAP = 8 cmH_2_O and *F*_IO2_ = 0.25. COPD, chronic obstructive pulmonary disease; CPAP, continuous positive airway pressure.

Figures 2, 3, 4, 5, 6, 7 show the examples of the dynamical responses of the model. Figures 2, 3 show the *P*_aO2_ and *P*_aCO2_ responses of the model in moderate COPD (stage 1) (γ = 1.35) and advanced COPD (stage 2) (γ = 1.6), respectively, with no oxygen supplementation (*F*_IO2_ = 0.21) and no CPAP treatment. As is shown, in Figure 2, *P*_aO2_ stabilizes around 65 mm Hg and *P*_aCO2_ settles around 46 mm Hg, indicative of relative hypoxemia and hypercapnia, in about 28 min. Figure 3 shows that in more advanced COPD, *P*_aO2_ settles around 59 mm Hg and *P*_aCO2_ stabilizes about 48 to 49 mm Hg in around 28 min indicative of more highly pronounced hypoxemia and hypercapnia.

Figure 4 shows the *P*_aO2_ and *P*_aCO2_ responses of the model in moderate COPD (stage 1) (γ = 1.35) with no oxygen supplementation and CPAP of 5 cmH_2_O. As is seen, *P*_aO2_ stabilizes around 65 mm Hg in about 28 min and *P*_aCO2_ is around 44 to 45 mm Hg indicative of hypoxemia and mild hypercapnia.

Figure 5 shows the arterial blood gas levels in moderate COPD (γ = 1.35) with CPAP at the same level of 5 cmH_2_O as in Figure 4, but *F*_IO2_ raised slightly to 0.25. As seen in the figure, *P*_aO2_ rises to about 93 mm Hg and *P*_aCO2_ is around 46 mm Hg in about 25 min indicative of improvement of the oxygen level compared with the results of Figure 4 resulting in normoxia, and mild hypercapnia.

Figure 6 shows the *P*_aO2_ and *P*_aCO2_ levels in advanced stage 2 COPD (γ = 1.6) with CPAP at 5 cmH_2_O, and F_IO2_ at 0.25. As shown in this figure, *P*_aO2_ reaches to about 87 to 91 mm Hg and *P*_aCO2_ stabilizes around 48 to 50 mm Hg in about 35 min. These results indicate normoxia and hypercapnia.

Figure 7 shows the arterial blood gas levels in advanced stage 2 COPD (γ = 1.6) with CPAP at 8 cmH_2_O, and F_IO2_ at 0.25. As is seen, *P*_aO2_ reaches to around 90 mm Hg and *P*_aCO2_ is about 50 mm Hg in about 35 min indicating normoxia and hypercapnia and no significant improvement in blood gases compared to Figure 6 in which CPAP was at 5 cmH_2_O.

## 4 DISCUSSION

It can be seen from the simulation results of Table 1 that the hypoxemia and hypercapnia are consistent with that observed in COPD patients breathing room air (*F*_IO2_ = 0.21) without any intervention, at moderate COPD (i.e. stage 1). This is also seen in simulation of more advanced stage of the disease (i.e. stage 2), with hypoxemia and hypercapnia becoming more highly pronounced as the disease advances. When low level of oxygen supplementation (i.e. *F*_IO2_ = 0.25) is used in moderate COPD, *P*_aO2_ increases to normal level (i.e. 90 mm Hg) with *P*_aCO2_ increasing slightly from 46 to 46.6 mm Hg. Application of a low level of CPAP of 5 cmH_2_O, without oxygen supplementation, does not show a significant improvement in blood gases. However, when CPAP of 5 cmH_2_O is combined with low level of oxygen supplementation (i.e. *F*_IO2_ = 0.25) *P*_aO2_ rises to 91 mm Hg and the rise in *P*_aCO2_ is due to carbon dioxide retention slightly decreasing.

The use of a higher level of CPAP does not significantly improve blood gases and the use of a higher level of oxygen supplementation (i.e. *F*_IO2_ = 0.3) causes more carbon dioxide retention accompanied by hyperoxemia. These results are in general agreement with clinical observations [15, 22]. The cardiac output and to a lesser extent the cerebral blood flow increase in response to lowered oxygen and elevated carbon dioxide levels in the blood in the modelled experiments. The minute ventilation rises to compensate for the rise in the dead space and in response to hypercapnia and hypoxemia, albeit to a lesser extent than that seen in normal individuals due to the shift in the acid–base balance of the patients. Due to the increased work of breathing and higher than normal airway resistance, the breathing rate remains low between 9.5 and 11.7 breaths per minute.

In simulation of more advanced COPD, lower level of *P*_aO2_ (i.e. 59.2 mm Hg) and a higher level of *P*_aCO2_ (i.e. 48.7 mm Hg) are observed in patients breathing room air without any CPAP. Use of low level of CPAP (i.e. CPAP = 5 cmH_2_O) improves *P*_aO2_ slightly to 62 mm Hg. Combining low CPAP of 5 cmH_2_O with low level of oxygen supplementation (*F*_IO2_ = 0.25) results in normoxia (*P*_aO2_ = 90.5 mm Hg), but it is accompanied by a slight elevation of *P*_aCO2_ from 48.57 to 50.16 mm Hg due to carbon dioxide retention. Higher level of CPAP (CPAP = 8 cmH_2_O) without oxygen supplementation results in *P*_aO2_ of about 60.5 mm Hg, and a higher level of *F*_IO2_ (*F*_IO2_ = 0.3) results in hyperoxemia and more pronounced hypercapnia.

The dynamic simulation results of Figures 2, 3, 4, 5, 6, 7 show the effects of CPAP therapy and oxygen supplementation in COPD patients in comparison with no intervention. These results show the persistence of hypercapnia at two stages of the disease and the effects of CO_2_ retention with oxygen supplementation that are exacerbated at the advanced stage of the illness. These results are seen to be in general agreement with clinical observations [11, 15, 22].

## 5 CONCLUSION

As a result of the damages to the alveoli that lead to alteration in the ventilation/perfusion ratio and inflammation of the bronchi, the work of breathing increases in COPD patients. The extra burden of the additional work of breathing results in inadequate ventilation over time, leading to progressive and persistent hypoxemia and hypercapnia, and rises in bicarbonate concentration in the blood and CSF. This chronic acid–base adjustment reduces the effects of CO_2_ as a stimulus to ventilation and causes what is referred to as ‘CO_2_ retention’, especially when oxygen supplementation is used in the patients to improve hypoxemia. The chronic hypoxemia and hypercapnia in COPD patients can have many adverse and lasting effects on their body organs and need to be mitigated for the patients’ bodies to overcome the disease conditions over time. CPAP therapy and oxygen supplementation provided by NIV are frequently used for COPD patients.

There have been recent studies to investigate the beneficial effects of long‐term home NIV treatment for COPD patients and suggestions have been made for optimization of NIV treatments in COPD to improve the treatment outcome (e.g. 17–18).

As the rate of COPD mortalities has increased significantly in recent years, there has been renewed interest in using simulation‐based optimization techniques to help with the treatment of this disease. We hypothesized that mathematical modelling of the respiratory system may be useful to help guide beneficial interventions for COPD patients using NIV. The results of this modelling suggest that under non‐emergency conditions, use of low level of CPAP alone can improve patient's oxygenation slightly without any significant increase in hypercapnia. The simulation results of the study predict that when a low level of *F*_IO2_ (e.g. *F*_IO2_ = 0.25) is combined with a low level of CPAP (e.g. CPAP = 5 cmH_2_O), the blood gases may improve significantly. The use of higher levels of CPAP did not improve blood gases significantly and the application of high *F*_IO2_ values was accompanied by undesirable CO_2_ retention. The results of this study suggest low levels of *F*_IO2_ (e.g. *F*_IO2_ = 0.25) combined with low levels of CPAP (e.g. CPAP = 5 cmH_2_O) over prolonged periods of time may be effective to counter the negative impacts of COPD on blood gases. The results of this study apply to moderate to more advanced COPD patients under non‐emergency conditions. Further studies including clinical applications of the proposed model may provide more information on the effectiveness and any limitations of the model as a guide for NIV treatment of COPD patients.

## AUTHOR CONTRIBUTIONS

Fleur Tehrani: methodology; mathematical modelling studies; literature search, writing original draft. James Roum: clinical evaluations; review and editing.

## CONFLICT OF INTEREST STATEMENT

The authors declare no conflict of interest.

## FUNDING INFORMATION

The authors received no funding for this work.

## Data Availability

The data that support the findings of this study are available from the corresponding author upon reasonable request.
